# The cardio‐respiratory effects of passive heating and the human thermoneutral zone

**DOI:** 10.14814/phy2.14973

**Published:** 2021-08-19

**Authors:** Mary E.T. Henderson, Daniel Brayson, Lewis G Halsey

**Affiliations:** ^1^ Centre for Research in Ecology, Evolution and Behaviour Life Sciences Department Whitelands College University of Roehampton London United Kingdom; ^2^ Dubowitz Neuromuscular Centre UCL, Great Ormond Street Institute of Child Health Holborn, London United Kingdom

**Keywords:** heat stress, human thermoneutral zone, hyperthermia, passive heating, resting metabolic rate

## Abstract

The thermoneutral zone (TNZ) defines the range of ambient temperatures at which resting metabolic rate (MR) is at a minimum. While the TNZ lower limit has been characterized, it is still unclear whether there is an upper limit, that is, beyond which MR during rest increases, and if so, what physiological upregulations explain this. We take the first step to fill this knowledge gap by measuring MR and multiple physiological variables in participants exposed to ambient heat stress while resting. Thirteen participants were exposed for an hour to 28℃‐50% relative humidity (RH) air, and both 40 and 50℃ each in 25% RH and humid (50% RH) conditions. Core and skin temperatures, blood pressure, sweat‐, heart‐, and breathing‐rate, minute ventilation, and movement levels were recorded throughout each condition. MR increased 35% (*p *= .015) during exposure to 40℃‐25% RH compared to baseline and a further 13% (*p *= .000) at in 50℃‐50%RH. This was not explained by increased fidgeting (*p *= .26), suggesting physiological upregulation. However, while greater heat stress invoked increases in heart rate (64%, *p *= .000), minute ventilation (78%, *p *= .000), and sweat rate (74%. *p *= .000) when comparing 50℃‐50% RH with baseline, the exact size of their relative energy cost is unclear and, therefore, so is their contribution to this increase in MR. Our study shows clear evidence that resting MR increases in humans at high temperature—there is a metabolic upper critical temperature, at least as low as 40℃. Further studies should pinpoint this value and fully explain this increased MR.

## Graphical abstract

Little research has been performed to characterize the upper human thermoneutral zone (TNZ). To find out if the resting metabolic rate (RMR) increases above the TNZ, 13 participants were exposed to a series of hot ambient temperatures. Our study found that RMR did, indeed, increase and, thus, is the first to provide evidence of a metabolic upper critical temperature.

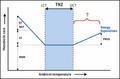

## 1 INTRODUCTION

Above an ambient temperature of 28℃, the metabolic processes of our body at rest produce sufficient heat to maintain our core's temperature at about the optimal 37℃ (IUPS, 2001; Jessen, 2001). Below about 28℃ (Brychta et al., 2019; Pallubinsky et al., 2019)—termed the lower critical temperature (LCT)—we need to expend more energy, to generate additional heat in order to maintain core temperature. Shivering thermogenesis is the key mechanism to achieve this, whereby our body rapidly contracts key muscle groups involuntarily (Gordon, 2012; Nowack et al., 2017). At high ambient temperatures, our body instigates alternate mechanisms to defend core temperature from temperature increases, including sweating and vasodilation of peripheral blood vessels.

**FIGURE 1:**
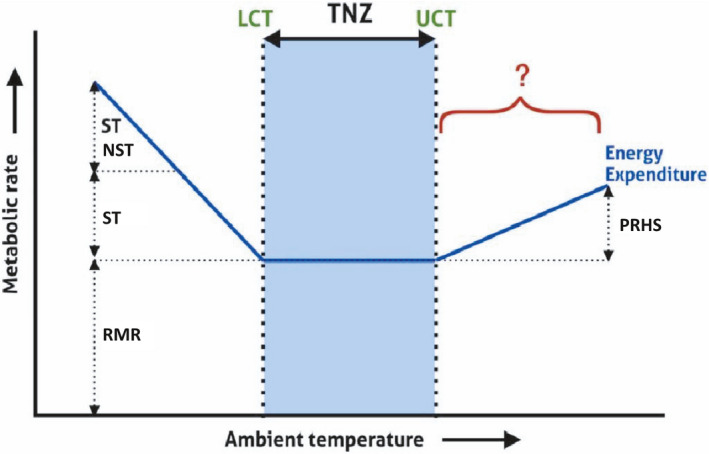
Schematic of the concept of the thermoneutral zone (TNZ), adapted from Pallubinsky et al. (2019). RMR: Resting metabolic rate; NST: non‐shivering thermogenesis; ST: shivering thermogenesis; LCT: lower critical temperature; UCT: upper critical temperature; PRHS: physiological responses to heat stress

Research on the human thermoneutral zone (TNZ), the range of ambient temperatures at which resting metabolism provides sufficient energy to maintain our core at 37℃, dates back to at least the 1930s (Hardy & Dubois, 1937, 1938). While the LCT is well characterized, a putative upper critical temperature (UCT) beyond which energy expenditure at rest increases has been far less explored. Indeed, it is unclear that a UCT for humans should exist, since neither sweating nor blood vessel vasodilation might be expected to be energetically costly (Weiner & Heyningen, 1952) (Figure 1).

Based on the observation that humans start sweating (thus generating evaporative heat loss) at around 32℃, Stolwijk and Hardy (1966) suggested that the thermoneutral zone of humans ranges from 28℃ to 32℃. However, they did not detect a rise in MR when exposing people for an hour to single ambient temperatures up to 48℃. Moreover, Pallubinsky et al. (2019) did not find evidence for an increase in MR with ambient temperature when exposing participants to a dynamic, passive heat acclimation culminating at 41℃. Both these studies were conducted on semi‐nude humans at relative humidity of around 26%. In contrast, however, Faerevik et al. (2001) observed an increase in MR at 40℃, at a similar relative humidity (RH). These three studies differ in their methods and primary research aims, which may explain their contrasting conclusions. Importantly, none of them controlled for the levels of fidgeting by the participants which, even at low levels, can drive up MR substantially (Levine et al., 1999), and thus, if fidgeting covaries with ambient temperature it could be the explanation in cases where increases in MR with temperature have been observed.

Consequently, the putative metabolic UCT remains unsubstantiated and, if present, the mechanisms responsible unexplained. In the current study, we investigated whether there are ambient temperatures at which resting MR in semi‐nude human beings increases, which, if so, would demonstrate that there must be a metabolic UCT. We also investigated the effect of dry (25% RH) and humid (50% RH) conditions on MR—a factor that could help to explain the conflicting findings of previous studies. Furthermore, we measured body movement, multiple body temperatures, sweat rate, and a range of cardio‐respiratory variables to investigate possible mechanisms explaining any observed increases in MR, and if and how these measures progress over time.

Previous studies investigating passive responses to heat employed either a ramped passive heating protocol or a series of discrete constant heat exposures (Stolwijk & Hardy, 1966 vs. Faerevik et al., 2001). Both approaches have advantages and disadvantages; a ramped protocol exposes participants to many temperatures but too briefly to account for physiological lags and also suffers from carry‐over effects, while a discrete protocol is less vulnerable to lags and can circumvent carry‐over effects but cannot pinpoint the temperature at which a response is first triggered. Given that our main aim is to assess if resting MR increases at a high temperature in general, as a first step to understanding the putative UCT of the human TNZ, we employed a discrete exposure protocol, setting treatment temperatures based on the literature.

## 2 MATERIALS AND METHODS

Thirteen healthy volunteers (seven female, mean age: 32.7 years ± 8.2 SD, range: 23–58 years; mean height: 171.5 cm ± 12.0 SD, 159–192 cm; mean body mass: 68.9 kg ± 13.8, SD 48.4–99 kg; mean body fat: 19.7% ± 7.6 SD, 11–42.7%) were recruited for this study. Participants were eligible if they met the following criteria: were aged 18–60 years, in good health (they were screened with health questionnaire SOP/LSC: 00591) and had not been on holiday to a hot country (30℃+) within the 3 weeks prior to starting the study (to avoid potential confounds of residual acclimatization). The core body temperature of females varies throughout their menstrual cycle so female participants were asked to participate in experiments during the first two cycle phases (Tenaglia et al., 1999). Before taking part, volunteers were given the participant information sheet which outlined the study aims and methods, and their informed consent was obtained. Ethical approval for the study protocol was granted by the ethics committee of the University of Roehampton (LSC:19/278). The study complied with the declaration of Helsinki (World Medical Association, 2013).

### 2.1 Study protocol

**FIGURE 2:**
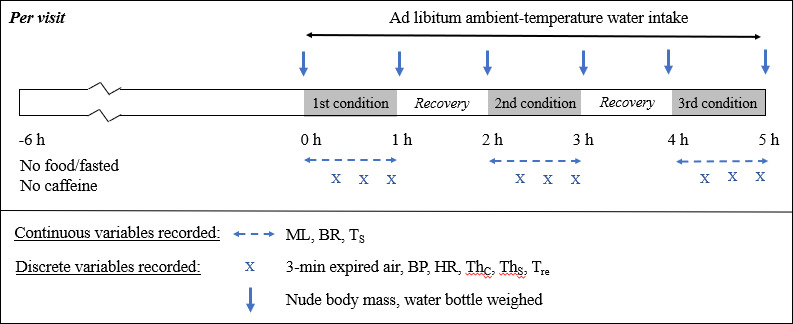
Study protocol per visit: each participant made three visits to the labs on three days. Each visit comprised of exposure to three randomized conditions each for 1 hour, with typically an hour recovery in between. BP: blood pressure; BR: breathing rate; ML: movement levels; Th_C_: thermal comfort; HR: heart rate; Th_S_: thermal sensation; T_s_: skin temperature; T_re_: rectal temperature

Participants attended the laboratories at Whitelands College, University of Roehampton, UK, on three separate visits for the experiments (Figure 2). These experiments investigated their physiological thermoregulatory responses during 1‐hour exposures in the environment chamber to five different ambient conditions defined by temperature and humidity. The participants outside the chamber between conditions for approximately 30 min or otherwise longer and until their core temperature had returned to the baseline measured at the beginning of each visit. The experimental conditions, except for the control condition, were repeated twice, thus participants experienced nine 1‐hour sessions, three per visit. Participants attended their visits at the same time of day to control for the effects of circadian rhythm on temperature regulation.

The baseline condition was 28℃‐50% RH. The other four conditions were combinations of 40℃ and 50℃, dry (25% RH), and humid (50% RH). The conditions were randomized for each participant while ensuring that participants did not undertake two conditions in a row at 50℃. Some participants undertook the experiments on their own, while other participants were in pairs or threes. Participant height (Harpenden Stadiometer, Holtain Ltd, UK), body mass (weighing scales, Seca, Birmingham, UK), and percentage body fat (%) via the method of whole‐body air displacement plethysmography (Wagner et al., 2000) (BodPod, COSMED, Italy) were measured during their first visit.

Participants were requested to abstain from alcohol and not to exercise for 48 h prior to each visit. They refrained from eating 6 h before and consuming caffeine 3 h before arrival. A small snack (~70 kcal) was given at the beginning of each 1‐hour condition to stave off hunger, calculated to minimize diet‐induced thermogenesis, without raising MR (Secor, 2009). Participants drank water that was of a temperature matching ambient conditions, ad libitum, which ranged between 27℃ and 34℃ at 40℃ ambient temperature, and between 30℃ and 42℃ at 50℃ ambient temperature over the duration of the condition. During the experiments, participants rested in a semi‐supine position on a stretcher made of air‐permeable material, wearing a breathable vest and shorts that standardised clothing to between 0.06 and 0.09 clo. A range of physiological measures was taken throughout the experiments to address the study aims as well as to monitor the safety of the participants.

### 2.2 Physiological measurements

The participants reported to the laboratory euhydrated (1–3 on the NHS hydration urine color chart; Armstrong et al., 1994). Participants self‐recorded nude body mass (BM) and self‐inserted a rectal thermistor (REC‐U‐VL3‐0, Grant Instruments Ltd., (Cambridge) Ltd., UK) ~10cm past the anal sphincter. A BioHarness (Zephyr Technology Corporation, Annapolis, MD, USA) was affixed to their upper torso. The BioHarness continuously recorded their movement levels (“vector magnitude units” calculated from the acceleration of the device measured in g; VMU = √(x^2^ + y^2^ + z^2^) where x, y, and z are the averages of the three axial acceleration magnitudes over the previous one) and breathing rates (again from the measures of acceleration; breaths.min^−1^). The rectal thermistor was connected to a portable data logger (Squirrel 2020 Series, Grant Instruments Ltd., Cambridge, UK), iButton skin temperature data loggers DS1922L, (Thermochron iButton, USA). iButtons were attached using transparent adhesive dressing (Tegaderm, 3 M Health Care, St Paul, MN) and waterproof tape (Transpore, 3 M Health Care, St Paul, MN) to the sternal notch, forearm, thigh, and calf muscle on the right side of the body. From the resulting data, mean‐weighted *T*_s_ was calculated (Ramanathan, 1964).

The blood pressure (mmHg) and heart rate (beats min^−1^) of each participant were recorded in triplicate after 20, 40, and 60 min with a digital sphygmomanometer (UA767, A&D Company Ltd., Tokyo, Japan) and averaged for each time point. Systolic blood pressure (SBP) and heart rate (HR) measurements were used to calculate rate pressure product (formula: SBP.HR (mmHg.bpm)) as a measure of myocardial workload. Three minutes of expired air samples were also collected at these time points, using the Douglas bag method, and subsequently analyzed (1400 series, Servomex, East Sussex, UK; Harvard Dry Gas Meter, Harvard Ltd., Kent, UK). Oxygen consumption (V̇O_2_) was calculated as a proxy for MR, O_2_, and CO_2_ measurements were corrected to the standard temperature and pressure and dry. The volume of water that each participant drunk during each condition was measured and they self‐recorded a final nude body mass measurement after they had towel dried. Sweat losses were determined from changes in body mass during the experiment, subtracting the weight of urine produced and adding fluid consumed (g). The participants were asked for their thermal comfort (Gagge et al., 1967) and thermal sensation scores (Young et al., 1987) at each time point for health and safety.

### 2.3 Data analysis

Full datasets for 13 participants were obtained for all measures except MR. Some MR calculations were erroneous and, therefore, full datasets for resting MR were obtained for seven participants and datasets for some conditions obtained for a further two participants. For comparing MR between conditions, we analyzed the seven participants, while correlations between MR and other measured variables were included data for nine participants. When testing for differences between conditions, the measurements taken at 60 min were compared. Data collected at all three‐time points were used to test for bivariate correlations between variables. Movement levels of the participant at each of the three‐time points per condition were derived by averaging a 4.5‐min time period beginning 90 s prior to the expired air collection, thus incorporating movement levels both immediately before and during the period of MR measurement. Breathing rates were determined by taking an average measure over 1.5 min while the respirometry mask was being worn and, separately, an average taken from three 1‐minute samples at 3, 4, and 5 min preceding mask wearing, enabling us to check for any effect of mask wearing on breathing frequency.

### 2.4 Statistical analysis

Statistical analyses were conducted using IBM SPSS statistical programming software (v26, SPSS). Differences between conditions and correlations between variables were analyzed using general linear mixed‐effects models to account for repeated measures within‐subjects, followed by least squared difference post hoc tests where appropriate. All data were tested for the necessary parametric assumptions and consequently, the movement data were transformed by square‐rooting the data to correct left‐skewness. The variables were considered sufficiently parametric to ensure robust interpretation from mixed‐effects linear models (Schielzeth et al., 2020). *p*‐values were calculated for these analyses and interpreted as providing some evidence for an effect when *p* < .05 and strong evidence for an effect when *p* < .01 (Benjamin et al., 2018). We use this to better qualify our results and their interpretation. We distinguish between those that only just achieve the *p* ≤ .05 significance level whereby a significant result that the null hypothesis is true can carry a 1 in 3 chance of being a “false positive” when factors that create “false positives” and other errors are accounted for; and the considerably lower significance level of *p* ≤ .01, whereby there is a far lower probability of the null hypothesis being true, as proposed by Benjamin et al. (2018).

## 3 RESULTS

Physiological and behavioral measures were obtained for 13 participants, while MR was not recorded for all participants on all conditions because the drierite within the respirometry system saturated unexpectedly quickly. The characteristics of the participants are as follows: mean age: 33.9 years ± 8.2 SD, range 28–41 years, mean height: 171.5 cm ± 14.0 SD, range 164–192 cm, mean body mass: 71.11 kg ± 17.7 SD, range 48.4–87.2 kg, mean body fat: 19.4% ± 10.1 SD, range 11–43%; Table 1.

**TABLE 1:** Physiological and behavioral measures of participants taken after 60 min of resting in various temperature–humidity conditions

Condition (℃/% RH)	28℃−50%RH	40℃−25%	40℃−50%	50℃−25%	50℃−50%
MR, L.min^−1^	**0.19 ± 0.05	*0.25 ± 0.05	0.24 ± 0.06	0.26 ± 0.05	*0.29 ± 0.04
ML, VMU	**0.02 ± 0.02	0.03 ± 0.03	0.02 ± 0.02	0.04 ± 0.05	** 0.03 ± 0.02
T_c,_ ℃	**36.82 ± 0.20	36.91 ± 0.24	37.04 ± 0.26	37.16 ± 0.41	** 37.88 ± 0.55
T_s,_ ℃	**34.66 ± 1.20	**36.92 ± 0.84	36.79 ± 0.34	**37.59 ± 0.78	** 38.84 ± 1.76
HR, bpm	**57.80 ± 9.28	**67.13 ± 10.18	67.29 ± 11.53	72.42 ± 16.19	**95.07 ± 19.88
DBP, mmHg	**62.56 ± 11.78	58.03 ± 6.90	55.08 ± 8.47	56.38 ± 7.03	** 48.33 ± 13.98
SBP, mmHg	110.19 ± 11.51	106.69 ± 13.25	105.72 ± 11.05	107.15 ± 3.10	100.70 ± 30.30
BRwm, breaths.min^−1^	14.96 ± 4.31	*14.82 ± 4.07	13.81 ± 3.83	13.22 ± 4.36	12.66 ± 4.23
BRm, breaths.min^−1^	**17.69 ± 5.90	16.05 ± 4.77	15.46 ± 4.17	14.91 ± 5.70	13.66 ± 5.36
V̇E, L.min^−1^	**7.00 ± 4.18	6.11 ± 3.33	6.31 ± 3.46	7.68 ± 5.73	** 12.92 ± 6.38
SR, g.h^−1^	**0.01 ± 0.12	0.27 ± 0.28	0.22 ± 0.21	0.38 ± 0.25	** 0.66 ± 0.43
RPP, mmHg.bpm	**6317.7 ± 2016.2	7081.1 ± 1425.6	7164.7 ± 1378.4	7788.5 ± 1457.0	**9939.3 ± 1456.9

MR: metabolic rate, T_c:_ core temperature, HR: heart rate; SBP: systolic blood pressure, DBP: diastolic blood pressure, T_S_: skin temperature, SR: sweat rate, ML: movement levels, VMU: vector magnitude unit, BRwm: breathing rate without respirometry mask, BRm: breathing rate with respirometry mask, V̇E: minute ventilation (volume of expired air per min), RPP: Rate pressure product.

Data are presented as mean ± SD and with a significance of *p* < .05* (some evidence) or *p* < .01** (strong evidence) for comparisons with the condition in the column to the immediate left, or with 50℃‐50% RH in the case of 28℃‐50% RH. *N* = 13 for all variables bar MR for which *N* = 7.

### 3.1 Metabolic rate

**FIGURE 3:**
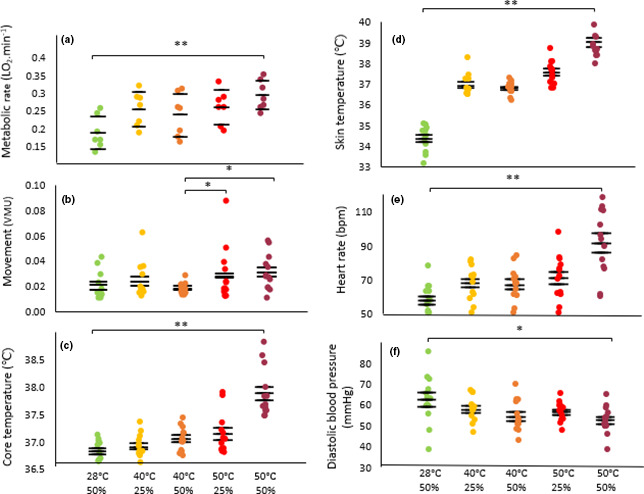
Changes in behavior and physiology in resting participants after 60 min of exposure to a control condition and various heat stress conditions. (a) Metabolic rate (*N* = 7, *n* = 35); (b) movement levels (VMU: Vector magnitude units. Static < 0.2; *N* = 13, *n* = 350); (c) core temperature (*N* = 13, *n* = 117); (d) skin temperature (*N* = 13, *n* = 117); (e) heart rate (*N* = 13, *n* = 115); (f) diastolic blood pressure (*N* = 13, *n* = 117), of resting participants in increasing heat stress conditions after 60 min. One data point per participant. The short horizontal bars denote the mean ± SD. Asterisks in combination with long horizontal bars denote significant differences between conditions: * *p* < .05, ** *p* < .01

Our experiments provide strong evidence that MR during rest increases in the highest heat stress conditions (F_4_ = 4.436, *p* = .006, *N *= 7, *n* = 35; Figure 3a). There is a particularly large increase between baseline and 40℃‐25% RH, and also between 50℃ −25% RH and the highest heat stress condition, 50℃‐50% RH. There is no evidence that levels of body movement vary between conditions nor that they covary with V̇O_2_ (F_4_ = 1.34, *p* = .260, *N* = 13, *n* = 350, Figure 3b).

### 3.2 Body temperature

**FIGURE 4:**
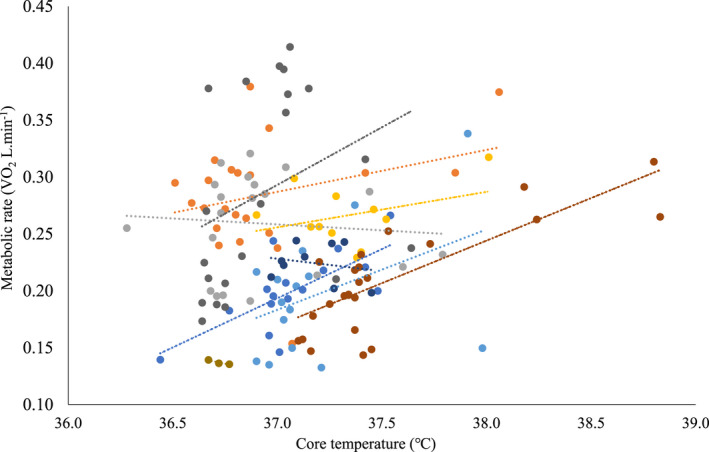
Metabolic rate against core temperature for participants during rest, across the control, and all heat stress conditions including the control (color coded by the participant). The stippled lines are the lines of best fit for each participant. *N* = 9, *n* = 149

**FIGURE 5:**
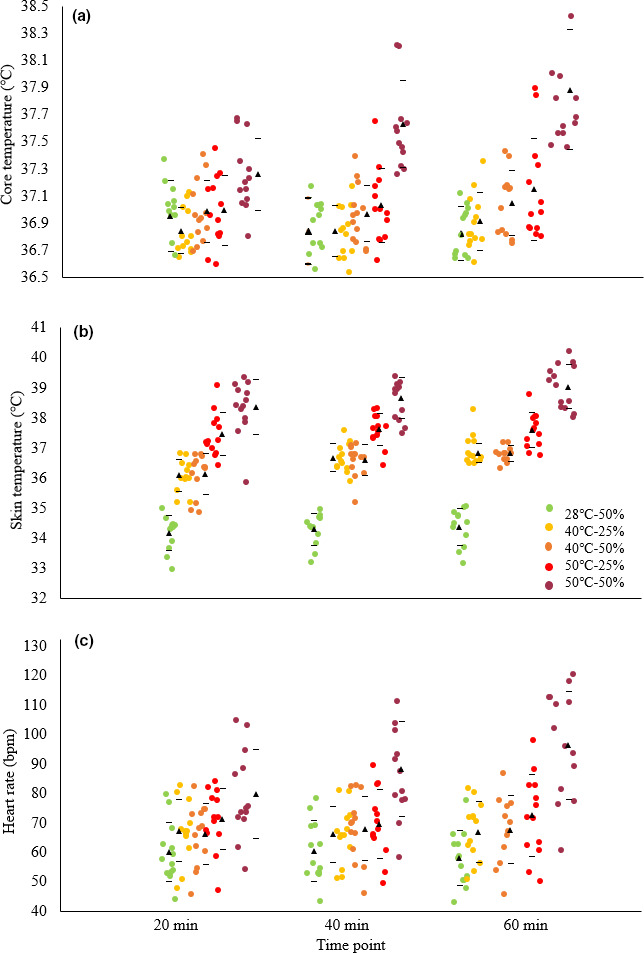
Changes in core temperature (a), skin temperature (b), and heart rate (c) over time for participants during rest in a control and various heat stress conditions. The triangles and horizontal bars represent the mean ± SD for each time point per condition. *N* = 13, *n* = 351

There is strong evidence of a higher core temperature in the participants when exposed to the higher heat stress conditions, most markedly at 50℃‐50% RH (F_4_ = 45.69, *p* = .000, *N* = 13, *n* = 117, Figure 3c). Moreover, core temperature correlates with MR (F_1_ = 15.20, *p* = .000, *N* = 9, *n* = 149, conditional *R* ^2^ = .49; Figure 4). Skin temperature also positively relates to heat stress (F_4_ = 31.07, *p* = .000, *N* = 13, *n* = 117, Figure 3d), responding to ambient conditions in the first 20 min of exposure and then remaining fairly constant for the rest of the 60 min (F_1_,_12_ = .21, *p* = .66; *n* = 351, *N* = 13; Figure 5b). In contrast, for core temperature there is a time–condition interaction whereby clear increases in core temperature are not observed until the measurements at 60 min (F_1_,_12_ = 53.60, *p* < .001, *N* = 13, *n* = 351; Figure 5a), and only in the conditions of 50℃‐25% RH (*p* = .000) and 50% RH (*p* = .000).

### 3.3 Sweat rate

There was some evidence that sweat rate increases in the highest heat stress conditions, 50℃‐25% RH and 50℃‐50% RH, compared to baseline (overall model: F_4_ = 2,47, *p* = .044, *N* = 13, *n* = 116; mean difference: .28 g.h^−1^, post hoc *p* = .000).

### 3.4 Cardiorespiratory variables

**FIGURE 6:**
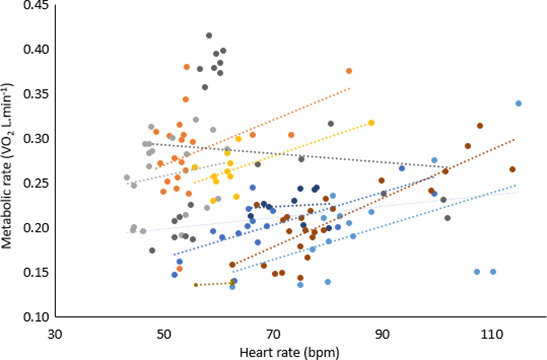
Metabolic rate against heart rate for nine participants during rest in all heat stress conditions including the control (color coded by the participant). The stippled lines are the lines of best fit for each participant. *N* = 9, *n* = 149

**FIGURE 7:**
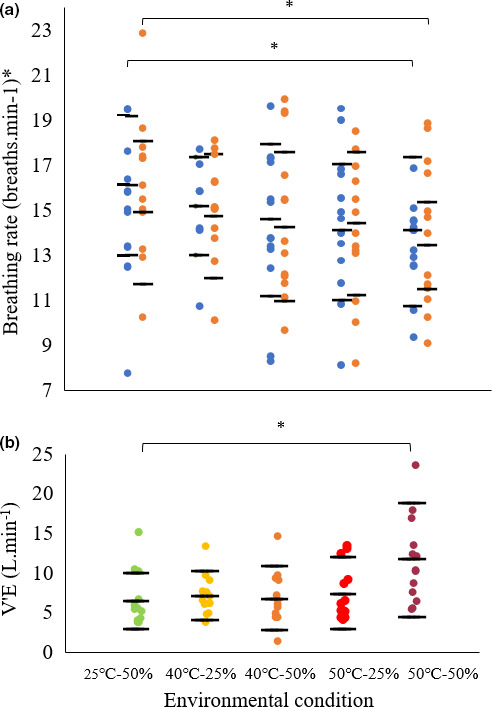
(a) Breathing rates of participants at rest in the control and each heat stress condition (blue dots: without respirometry mask; orange dots: wearing respirometry mask). *N* = 13, *n* = 116. (b) Volume of expired air against heat stress condition. One data point per participant. The horizontal bars denote the mean ± SD. Asterisks in combination with horizontal bars denote significant differences between conditions: * *p* < .05, ** *p* < .01. *N* = 13, *n* = 350

Changes in heart rate and blood pressure track the step change increases in MR at 40℃‐25% RH and 50℃‐50% RH (Figure 3e, f, and a, respectively). There is strong evidence that heart rate increases with increasing heat stress, by 16% at 40℃‐25% RH and 64% at 50℃‐50% RH compared to baseline (F_4_ = 45.16, *p* = .000, *N* = 13, *n* = 115; Figure 3e), and also that it correlates with MR (conditional *R* ^2^ = .45, *p* = .000, *N* = 9, *n* = 149; Figure 6). Diastolic blood pressure decreases as heat stress increases, displaying a 7% decrease of at 40℃‐25% RH and 23% at 50℃‐50% RH compared to baseline (F_4_ = 6.29, *p* = .000; *N* = 13, *n* = 117; Figure 3f). Systolic blood pressure did not differ between conditions (F_4_ = 1.01, *p* = .404, *N* = 13, *n* = 117). Rate pressure product showed a considerable increase at 40℃‐25% RH from a baseline of ~12% (763.3 mmHg ± 2469.4) and a further marked rise of 26% (1610.8 mmHg*bpm ± 2908.0) between 50℃‐25% RH and 50℃‐50% RH (F_4_ = 7.04, *p* = .000; *N* = 13, *n* = 349). There is some evidence that the breathing rate decreases with increasing heat stress, by 22.8% across all conditions from 17.7 to 13.7 breaths.min^−1^ (F_12_ = 2.25, *p* = .014, *N* = 13, *n* = 116, Figure 7a). However, there is strong evidence that the volume of air expired by the participants per minute increases with increasing heat stress (F_4_ = 9.41, *p* = .000, *N* = 13, *n* = 350, Figure 7b) with a rise of 78% between baseline and 50℃‐50% RH (*p* = .000). There is no evidence of a difference in the breathing rates of participants when wearing or not wearing a respirometry mask (F_1_ = 1.34, *p* = .25, *N* = 13, *n* = 350; Figure 7a).

## 4 DISCUSSION

**FIGURE 8:**
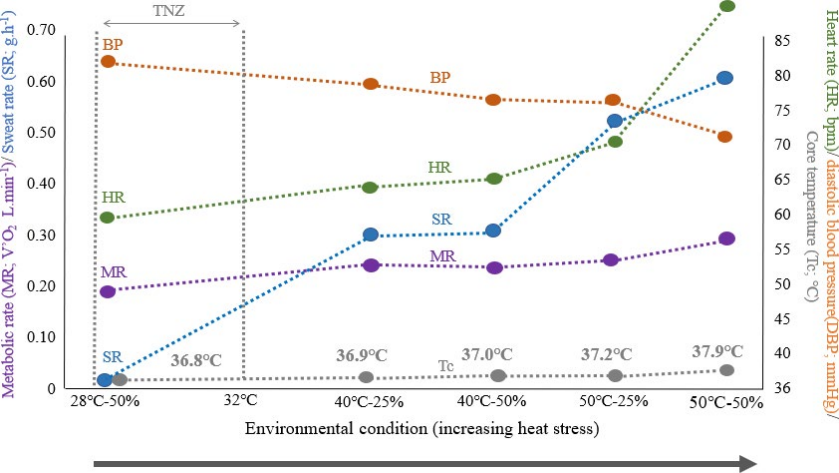
Summary of key changes in physiological variables measured in humans at rest in various heat stress conditions described by temperature and humidity

We observed a statistically significant and large (35%) increase in MR from baseline for resting, semi‐nude participants when exposed to 40℃‐25% RH air. In this condition, this increase in MR is not accompanied by an increase in core temperature (T_re_), suggesting that our participants are still able to fully dissipate heat (Figure 8). While our findings are in contrast to those of Stolwijk and Hardy (1966) and Pallubinsky et al. (2019), neither of which found an increase in MR at 48℃ and 41℃, respectively, this contrast might be explained by the much shorter exposure times for the participants in those two studies. Faerevik et al., (2001) found a similar increase in MR to that in the present study (23%) in participants exposed to air at 40℃‐25% RH for an hour. In Pearson et al.,’s study (2011) looking at hemodynamic responses to heat stress in the resting human leg, they observed that increases in core temperature >1℃ cause a 70% rise in absolute metabolic requirements of the whole body equating to an elevation in O_2_ consumption of ~0.15 L/min. Importantly, increases in MR in our study were not accompanied by increases in movement levels (Figure 3b), indicating that the former is driven by physiological adjustments. Thus, we conclude that the human TNZ does have an upper critical temperature, that is, above which MR at rest increases, and that this is at least as low as 40℃.

At 50℃‐25% RH, there was effectively no increase in MR over that at 40℃‐25% RH. However, in humid conditions at 50℃, MR is 13% higher at 50℃‐50% RH than at 50℃‐25% RH (and 56% higher than baseline). It is only at 50℃‐50% RH that we observe a rise in T_re_, of 1℃, indicating that the body is no longer able to dissipate sufficient heat to maintain its core temperature. No cooling effect of the water drunk by the participants was evident across the conditions; the difference in temperature between the water and core body temperature was consistent at each condition. In contrast, MR varied little between 25% RH and 50% RH air at 40℃. A possible explanation for this interaction effect between temperature and humidity on MR is that the increase in humidity between 40℃‐25% RH and 40℃‐50% RH is not sufficient to stifle heat dissipation by the body, whereas at 50℃, the increase in humidity between 25% and 50% RH substantially limits evaporative heat loss via sweating. Our participants sweated substantially more (74%) in the 50℃‐50% RH compared to 50℃‐50% RH, while there is no evidence for a difference in their sweat rates between 40℃‐50% and 40℃‐25% RH.

Many of the physiological variables we measured increased in value over the course of each 1‐hour exposure to heat stress. Where core temperature was recorded as increasing 1 hour after exposure, it only started to increase after about 40 min, with further increases by 60 min. In contrast, at 50℃‐50% RH, the heart rate increased much sooner into the exposure, that is, after at most 20 min, and continued to increase until the end of the exposure. Skin temperature, moreover, changed quickly and then remained fairly constant relative to its measurement after 20 min, in all conditions. Thus, variable measurements taken only at the end of the exposure period belie dynamic differences in how those variables change over time to exposure. Had the exposure times to certain temperatures been somewhat shorter than 1 hour, the effect sizes for many of the variables we measured would be reported as being somewhat smaller. In turn, even longer exposures may have reported greater effect sizes for certain variables. Differences in exposure time are a likely cause of different interpretations in the literature about the presence and nature of increases in resting MR at high temperatures.

### 4.1 Possible mechanisms responsible for an increase in MR with increasing heat stress Q_10_ Effect

The Q_10_ effect is defined as “the ratio of the rate of a physiological process at a particular temperature to the rate at a temperature 10℃ lower” (IUPS Thermal Commission, 2003). At higher temperatures, cellular components have increased kinetic energy which speeds up biochemical processes (Gillooly et al., 2001). This results in higher rates of physiological and sometimes also behavioral processes, generating greater energy consumption (Clarke & Fraser, 2004). *Q*_10_ = 2 is applied in human thermoregulation models (Fiala et al., 2012; Werner & Buse, 1988)—a value supported by the work of Kampmann and Bröde (2015) who looked at the influence of core temperature on oxygen uptake in acclimated males during exercise and as a result developed the following equation:

(1) $$\%MR=\left(Q_{10}^{\Delta Tc/10}‐1\right)\times 100\left(2019\right).$$

Using their equation (1), the average *Q*_10_ they report of 2.1 translates into a 7% increase in MR per 1℃ rise in *T*_C_. However, in the present study, we observed a mean increase in MR of 56.4% between ~1℃ increase in T_c_ between baseline and the 50℃‐50% RH condition. Therefore, at best the Q_10_ effect explains only a small part of the increase in MR that we observed.

### 4.2 Cardiac work

We observed mean increases in heart rate (HR) of 37.3 bpm between baseline and 50℃‐50% RH, which is in line with previous work (International Organisation for Standardisation (ISO), 2004; Kampmann, 2000; Kuhlemeier & Miller, 1978; Vogt et al., 1973; Bröde & Kampmann, 2019). We also observed an increase in myocardial workload (rate pressure product) of ~12% from baseline to 40℃‐25% RH and of ~26% from 50℃‐25% RH to 50℃‐50% RH. The physiological basis of this increase is due to a thermally mediated peripheral vasodilation and reduction in blood pressure leading to baroreceptor activation and sympathetic stimulation of heart rate. The skin vasculature receives 5–10% of cardiac output in normothermic conditions at rest. Some studies have found that when the blood vessels are fully dilated in response to thermal stress, the skin vasculature receives up to 6–8 L/min which accounts for 50–70% of cardiac output, (Johnson & Proppe, 1996; Rowell, 1974, 1977, 1983) of which active vasodilation is responsible for 80–95% of this change (Johnson & Proppe, 1996; Rowell, 1986). Alongside force of contraction, heart rate is the most important determinant of myocardial oxygen consumption (Crandall & Wilson, 2015; Chiesa et al., 2019; Boyette & Manna, 2019), and possibly accounts for a substantial increase in V̇O_2_ observed in high heat stress conditions in our participants.

### 4.3 Breathing

At 50℃‐50% RH, while breathing rate decreased by 23% compared to baseline, minute ventilation increased by 78% accompanied by a ~1℃ increase in Tc—when heat stressed, participants breathed a little more slowly but evidently more deeply. These findings concur with previous work showing that hyperpnea and elevated minute ventilation are hyperthermic responses in humans at rest when Tc rises by more than 1℃ (White, 2006; Saxton, 1981; Cabanac & White, 1995; Fujii et al., 2015).

Possibly, this response is to aid brain cooling through respiratory heat loss (White, 2006). Alternatively, raised body temperature could mediate this change in ventilation, through direct action on the central respiratory pacemaker (Cooper & Veale, 2011), delivering more oxygen to the body to support the increased MR. Breathing represents only 2% of resting MR in healthy normothermic humans (Barlett et al., 1958; Lounsbury et al., 2002; Otis, 1954). A metabolic cost of 0.35 ml of oxygen has been calculated for each liter increase in minute ventilation (Campbell et al., 1959). Applying this to the present data would only account for 2.1 ml.min^−1^ or ~2% of the increase in MR at 50℃‐50% RH compared to baseline. This observation concurs with the more recent work of Zakynthinos and Roussos (1991) who estimated the oxygen cost of breathing from changes in total‐body V̇O_2_. Applying their approach, the ~5L.min^−1^ increase in V̇E measured in our study between baseline and the most heat stressful condition translates into a 3.4% increase in V̇O_2_ of the respiratory muscles.

In conclusion, the increase in the metabolic rate in the heat seen in our study is probably the product of upregulations in multiple physiological systems, some representing a greater contribution than others, those perhaps being increased cardiac output. We measured both an increase in cardiac output (rate pressure product) and a fall in diastolic pressure, an indicator of vasodilation. To a lesser degree, the greater volume of air expired also incurs a greater energy cost, as explained above. This was not due to a faster breathing rate, as measured in our study, but to deeper more effortful breathing.

The findings of this study are of increasing importance for metabolic studies, particularly in the light of climate change and the increasing risk for overheating in the built environment (European Environment Agency (EEA), 2015).

## 5 STUDY LIMITATIONS

The length of exposure time to each experimental condition was constrained to 1 hour, although this is as long as any previous and similar protocol (Faerevik et al., 2001; Hasan & Niemi, 1954; Mitchell et al., 1968); to expose participants to longer at 50℃ would be onerous. While we observed substantial changes in a number of measured variables over the hour, it is quite possible that further changes would be observed over a more extended period. This raises an interesting question as to whether the definition of the TNZ should include a time component. Our study did not measure all conceivable cardiometabolic processes of relevance, such as cardiac output, and, therefore, the relative contribution of these processes remains to be determined. Future studies could include simultaneous echocardiographic assessment. While a larger sample size to compare MR between conditions would have been preferable, the effect size between certain conditions in our study is very substantial providing clear evidence of energy‐demanding physiological responses.

## 6 CONCLUSIONS

Resting, semi‐naked humans experience an increase in MR at ambient temperatures at least as low as 40℃‐25% RH air, not explained by changes in low‐level activity and not confounded by carry‐over effects from previous conditions. This is strong evidence for an increase in MR in resting humans when, at least abruptly, they are exposed to high ambient temperatures, and in turn that the human TNZ has an upper critical temperature. Testing responses to a series of discrete temperatures between 32 and 40℃ would uncover where the UCT lies on average and its between‐individual variability. The physiological changes that underpin these observed metabolic increases above the TNZ are at best only partially explained by the Q_10_ effect and upregulation of the cardio‐respiratory system, and require further investigation.

## DATA AVAILABILITY

The authors confirm that the data supporting the findings of this study are available within the article.

## CONFLICT OF INTEREST

All authors declared that there are no competing interests.

## AUTHOR CONTRIBUTIONS

All authors:

- contributed to the conception or design of the work,
- acquisition, analysis or interpretation of data for the work,
- drafting the work or revising it critically for important intellectual content,
- approved the final version of the manuscript,
- agreed to be accountable for all aspects of the work in ensuring that questions related to the accuracy or integrity of any part of the work are appropriately investigated and resolved,
- qualify for authorship, and all those who qualify for authorship are listed.
